# Long non-coding RNA H19 regulates matrisome signature and impacts cell behavior on MSC-engineered extracellular matrices

**DOI:** 10.1186/s13287-023-03250-6

**Published:** 2023-03-08

**Authors:** Sara Reis Moura, Jaime Freitas, Cláudia Ribeiro-Machado, Jorge Lopes, Nuno Neves, Helena Canhão, Ana Maria Rodrigues, Mário Adolfo Barbosa, Maria Inês Almeida

**Affiliations:** 1 Instituto de Investigação E Inovação Em Saúde (i3S), Universidade Do Porto, Rua Alfredo Allen, 208, 4200-135 Porto, Portugal; 2 Instituto de Engenharia Biomédica (INEB), Universidade Do Porto, Porto, Portugal; 3 Instituto de Ciências Biomédicas Abel Salazar (ICBAS), Universidade Do Porto, Porto, Portugal; 4 Departamento de Ortopedia, Centro Hospitalar Universitário São João (CHUSJ), Porto, Portugal; 5 Hospital CUF, Porto, Portugal; 6 Faculdade de Medicina (FMUP), Universidade Do Porto, Porto, Portugal; 7 NOVA Medical School - Faculdade de Ciências Médicas, Universidade Nova de Lisboa, Lisbon, Portugal; 8 Comprehensive Health Research Center (CHRC), Universidade Nova de Lisboa, Lisbon, Portugal

**Keywords:** Non-coding RNAs, Fragility fractures, Bone extracellular matrix, Gene therapy, Decellularization

## Abstract

**Background:**

The vast and promising class of long non-coding RNAs (lncRNAs) has been under investigation for distinct therapeutic applications. Nevertheless, their role as molecular drivers of bone regeneration remains poorly studied. The lncRNA *H19* mediates osteogenic differentiation of Mesenchymal Stem/Stromal Cells (MSCs) through the control of intracellular pathways. However, the effect of *H19* on the extracellular matrix (ECM) components is still largely unknown. This research study was designed to decode the *H19*-mediated ECM regulatory network, and to reveal how the decellularized siH19-engineered matrices influence MSC proliferation and fate. This is particularly relevant for diseases in which the ECM regulation and remodeling processes are disrupted, such as osteoporosis.

**Methods:**

Mass spectrometry-based quantitative proteomics analysis was used to identify ECM components, after oligonucleotides delivery to osteoporosis-derived hMSCs. Moreover, qRT-PCR, immunofluorescence and proliferation, differentiation and apoptosis assays were performed. Engineered matrices were decellularized, characterized by atomic force microscopy and repopulated with hMSC and pre-adipocytes. Clinical bone samples were characterized by histomorphometry analysis.

**Results:**

Our study provides an in-depth proteome-wide and matrisome-specific analysis of the ECM proteins controlled by the lncRNA *H19*. Using bone marrow-isolated MSC from patients with osteoporosis, we identified fibrillin-1 (FBN1), vitronectin (VTN) and collagen triple helix repeat containing 1 (CTHRC1), among others, as having different pattern levels following *H19* silencing. Decellularized siH19-engineered matrices are less dense and have a decreased collagen content compared with control matrices. Repopulation with naïve MSCs promotes a shift towards the adipogenic lineage in detriment of the osteogenic lineage and inhibits proliferation. In pre-adipocytes, these siH19-matrices enhance lipid droplets formation. Mechanistically, *H19* is targeted by miR-29c, whose expression is decreased in osteoporotic bone clinical samples. Accordingly, miR-29c impacts MSC proliferation and collagen production, but does not influence ALP staining or mineralization, revealing that *H19* silencing and miR-29c mimics have complementary but not overlapping functions.

**Conclusion:**

Our data suggest *H19* as a therapeutic target to engineer the bone ECM and to control cell behavior.

**Supplementary Information:**

The online version contains supplementary material available at 10.1186/s13287-023-03250-6.

## Introduction

Osteoporosis is a disease that affects more than 200 million people worldwide [1, 2]. It is a skeletal disease defined by a low bone mineral density (BMD) that results in the deterioration of the bone tissue microarchitecture/quality [1, 2]. It increases bone fragility and vulnerability to fractures, which is the most important cause of morbidity and loss of life quality in patients suffering from this disease [3–6]. Although BMD measurements are widely used as a diagnostic tool for osteoporosis, it is unrealistic to predict the risk of fracture merely based on changes in the BMD [7, 8]. Therefore, it is crucial to emphasize the importance of the use of other indicators of bone health [7, 8]. Among other properties related to bone quality, bone composition, the biophysical properties and structure of these components should be taken into consideration. In fact, the impairment of the composition of the organic portion of the bone was reported in animal models (e.g., DMP-1) [9, 10] and patients (e.g., collagens and osteocalcin (OCN)) [11–13] with osteoporosis, as well as a structural/orientational alteration of the fibrils [10, 14, 15]. These changes impact the matrix assembly and, consequently, the structural organization of the bone, influencing the mechanical properties, widely described to be impaired [9, 16, 17].

At the cellular level, osteoporosis is caused by a disruption in the homeostasis between bone formation and resorption [5, 18, 19], leading to the consequent degradation of the bone matrix. Recent studies have shown that the disruption of the commitment into the osteogenic lineage in benefit of adipogenesis may also be associated with disease progression and/or ageing, leading to a disturbance in bone regeneration/repair [20–23].

Thus, it is crucial to determine the role of key molecular regulators of Mesenchymal Stem/Stromal Cells (MSCs) differentiation and bone extracellular matrix (ECM) components for the development of new targeted therapies in osteoporosis and fragility fractures.

Long non-coding RNAs (lncRNAs) comprise a large class of non-protein-coding genes that regulate cellular mechanisms, including bone formation, remodeling and homeostasis [24–26]. Their ability to regulate bone-related mechanisms, as well as their abnormal expression in bone disorders, like osteoporosis, illustrates their potential as therapeutic tools and/or biomarkers [6, 27]. *H19* is an imprinted human lncRNA lacking an evolutionarily conserved open reading frame, but with a conserved secondary RNA structure, that has been described as a regulator of osteogenic differentiation [6, 28, 29]. Moreover, *H19* overexpression promotes heterotopic bone formation in vivo [28, 30, 31]. It works as an endogenous competing RNA by targeting mi(cro)RNAs (eg. miR-141 and miR-22 [28], miR-138 [32], miR-188 [33]), being able to decrease HDAC4/5 and TGF-β1 [30], activate the Wnt/β-catenin pathway [28], increase SDF-1 [29] and regulate FAK expression [32] in MSCs. Furthermore, *H19* is expressed at low levels in disuse osteoporosis [34]. Although several studies have shown that *H19* is able to modulate different intracellular mechanisms in MSCs, the impact on the ECM production and secretion, and on the ECM-cell interactions, is still poorly explored. On the other hand, the use of genetically engineered cells, enabling the production of pro-regenerative matrices, has emerged as an alternative approach in the field of regenerative medicine. Thus, understanding the regulators of ECM components, as well as bone cells behavior on engineered-cell-derived matrices, is crucial.

In this study, we aim to investigate the role of *H19* in ECM composition using osteoporosis-derived MSCs and to determine the impact of *H19*-engineered matrices on naïve MSCs and pre-adipocytes behavior.

## Materials and methods

### Clinical samples: bone tissue and human primary bone marrow-derived MSCs

Bone samples (*N* = 17) were collected at Centro Hospitalar Universitário São João (CHUSJ, Porto, Portugal) from discarded bone of patients that underwent hip surgery following a fragility fracture (FF). Human primary MSCs were isolated from the bone marrow derived from the femoral head of patients with fragility fractures (*N* = 6), following a previously described protocol [35]. MSCs from healthy donors (*N* = 4) were also collected from the tibia following surgery to anterior cruciate ligament injury. Patients with other medical conditions, such as tumors or inflammatory diseases, were excluded from the study. Clinicopathological data are provided in Additional file 1: Table SI (Additional file 1). Culture of MSCs is detailed in the Additional file 1: Methods section. The protocol was approved by the CHUSJ Ethics Committee for Health, according to the declaration of Helsinki, and signed informed consents were obtained from the study participants. RNA samples extracted from the trabecular bone at the distal extremity of the femoral epiphysis of patients with fragility fractures (*N* = 11) or osteoarthritis (*N* = 9) were obtained from the biobank at Instituto de Medicina Molecular (iMM, Lisbon Academic Medical Center, Lisbon, Portugal).

### Osteogenic and adipogenic differentiation of bone marrow-derived MSCs

To induce osteogenic differentiation, MSCs were incubated in the presence of osteogenic supplements: 10^–7^ M dexamethasone, 10^–2^ M β-glycerophosphate and 5 × 10^–5^ M ascorbic acid, all from Sigma-Aldrich. To induce adipogenic differentiation, bone marrow-derived MSCs were cultured in the presence of adipogenic supplements: 10^–4^ M dexamethasone, 10^–1^ mM Indomethacin, 5 × 10^–4^ M 3-Isobutyl-1-methylxanthine (IBMX) and 10 μg/mL insulin, all from Sigma-Aldrich. Cells were incubated under basal or osteogenic/adipogenic conditions, and the media was changed twice a week. To evaluate osteogenic differentiation, MSCs were fixed with 40 g/L paraformaldehyde (PFA, Sigma-Aldrich) for 30 min, at room temperature (RT), before the ALP and Alizarin stainings were performed (Additional file 1: Methods). To confirm adipogenic differentiation, cells were fixed with 40 g/L PFA and stained with Oil Red O solution (Additional file 1: Methods).

### RNA isolation, reverse transcription and real-time quantitative polymerase chain reaction (RT-qPCR)

RNA was isolated using TRIzol Reagent (Invitrogen), according to the manufacturer’s instructions. RNA concentration and purity were determined by measuring absorbance using a NanoDrop Spectrophotometer ND-1000 (Thermo Fisher Scientific). RNA integrity was evaluated by agarose gel electrophoresis or Agilent RNA 6 000 Pico kit (Agilent).

To test the expression of coding and long non-coding transcripts, total RNA was first treated with TURBO DNA-free kit (Life Technologies), following the manufacturer’s protocol, to remove potential DNA contaminants. RNA samples were then used to synthesize cDNA, using random hexamers (Life Technologies) and SuperScript® III Reverse Transcriptase kit (Life Technologies). qPCR reactions were performed using cDNA, iQ SYBR Green Supermix (Bio-Rad) and respective primers, according to the following conditions: 3 min at 95 °C and forty cycles of 30 s at 95 °C, 30 s at 58 °C and 30 s at 72 °C. β-actin was used as a reference gene. Primer sequences are described in Additional file 1: Table SII.

To test the expression of miRNAs, TaqMan miRNA assays (Applied Biosystems) were used, according to the manufacturer’s protocol. Briefly, complementary DNA (cDNA) was synthesized using total RNA, TaqMan MicroRNA Reverse Transcription Kit and gene specific stem-loop Reverse Transcription primers (hsa-miR-29a-3p. hsa-miR-29b-3p and hsa-miR-29c-3p, Applied Biosystems; Additional file 1: Table SIII). qPCR was carried out in a thermal cycler (MyCycler Thermal Cycler, Bio-Rad) using cDNA, SsoAdvanced™ Universal Probes Supermix (Bio-Rad) and TaqMan probes (Applied Biosystems), according to the following conditions: 10 min at 95 °C, 40 cycles of 15 s at 95 °C and 1 min at 60 °C. Small nuclear RNA U6 was used as a reference gene.

All RT-qPCR reactions were performed in duplicate and data was analyzed using Bio-Rad CFX Manager software (Bio-Rad). Relative expression levels were calculated using the quantification cycle (Ct) method, according to MIQE guidelines [36].

### In silico target predictions

miRNA FASTA format sequence annotations were obtained from the miRBase database (http://www.mirbase.org/). In silico predictions of the interactions between miRNAs with lncRNAs were performed using Encyclopedia of RNA Interactomes (ENCORI).

### MSC transfection

For functional assays, MSCs were transfected with a small interference RNA against *H19* (20 nM; silencer select pre-designed siRNA for *H19*; siH19-1, also named siH19, Assay ID: n548065; siH19-2, Assay ID: n272453) or the respective control (20 nM; silencer select negative control; siCTR); or with miR-29c-3p mimics (50 nM; mimics), miR-29c-3p inhibitor (50 nM; inhibitor) or the respective controls (50 nM; miRNA mimics negative control and miRNA inhibitors negative control), all from Ambion. Oligonucleotide transfection was performed using Lipofectamine 2 000 reagent (Invitrogen), according to manufacturer’s protocol. MSCs were plated the day before the transfection at a cell density of 0.02 × 10^6^ cells/cm^2^ and incubated for 12 h with the oligonucleotides-liposomal complexes in media without penicillin/streptomycin (P/S, Invitrogen). Cells were cultured with basal growth or osteogenic media, depending on the assay.

### Protein isolation and identification by nanoscale liquid chromatography coupled to tandem mass spectrometry (nano LC–MS/MS)

Proteins were isolated from siH19-MSCs or respective control using an extraction buffer for matrix proteins enrichment, following 7 days of culture in osteogenic-inducing conditions. Briefly, cells were washed four times with cold PBS 1 × and a 2 M urea solution [4% (v/v) SDS (Sigma-Aldrich), 60 mM Tris–HCl (Sigma-Aldrich), 1 mM EDTA (Sigma-Aldrich); pH = 6.8] was used to solubilize the ECM and lysate the cells in the presence of protease (1:100, Thermo Fisher Scientific) and phosphatase inhibitors (1:100, Thermo Fisher Scientific). The solution was added directly to the cell culture plate to avoid losing ECM and culture plates were incubated for 30 min under constant rotation. The protein was collected to low binding microcentrifuge tubes (Eppendorf) and the lysates/solubilized ECM were spun down at 16 000 g for 5 min. Total protein concentration was quantified using the DC protein assay kit (Bio-Rad).

Protein identification and quantitation was performed by nano LC–MS/MS, using an equipment composed by an Ultimate 3000 liquid chromatography system coupled to a Q-Exactive Hybrid Quadrupole-Orbitrap mass spectrometer (Thermo Fisher Scientific), as previously described [37]. Further details are provided in Additional file 1: Methods. Bioinformatic and data analysis were performed for proteins expressed in three independent experiments with fold-change (FC) differences ≥ 1.25 or ≤  − 1.25 and *p* < 0.05. A minimum of two unique peptides was required for further evaluation. The differentially expressed proteins were classified according to gene ontology (GO) annotations. The enriched biological process analysis was performed using Protein ANalysis THrough Evolutionary Relationships (PANTHER) tool. Pathway analysis was performed using the software Ingenuity Pathway Analysis (IPA). For target validation, proteins were selected according to the following criteria: more than four unique peptides that simultaneously associate with cellular terms “non-structural extracellular” and “extracellular matrix” (*p*-value < 0.01) and with the molecular function “extracellular structural activity”.

### Immunocytochemistry

MSCs were washed and fixed with 40 g/L PFA for 30 min at RT. Cells were permeabilized with 0.1% (v/v) Triton X‐100 (Sigma-Aldrich) for 10 min, at RT, rinsed and blocked with 5 g/L BSA/ 0.1% (v/v) Triton X‐100 for 1 h at RT. Then, cells were incubated with primary antibody against collagen type I (COL1, 1:250, Rockland), osteopontin (OPN; 1:250, Santa Cruz Biotechnology), fibrillin-1 (FBN1; 1:100, Thermo Fisher Scientific), collagen Triple Helix Repeat Containing 1 (CTHRC1; 1:50, Santa Cruz Biotechnology) and vitronectin (VTN; 1:25, Santa Cruz Biotechnology) overnight at 4 °C. Cells were washed three times with PBS 1x, 5 min each, incubated with the respective secondary antibody (1:1 000; Thermo Fisher Scientific) for 1 h at RT and washed again. Cell nuclei were stained with 1 μg/mL DAPI (Invitrogen) for 5 min. The antibodies manufacturers and respective dilutions are detailed in Additional file 1: Table SIV. The stainings were visualized under the Leica DMi6000 FFW (Leica Microsystems). Cells incubated with the antibody diluent alone (without primary antibody), followed by incubation with the secondary antibody, were used as negative controls. Semi-quantification of the protein was performed using the ImageJ Fiji software, considering a minimum of 6 distinct fields per condition.

### Resazurin reduction assay

MSCs were seeded with a cell density of 2 × 10^3^ cells/well in 96-well plates. The resazurin redox dye (0.01 mg/mL, Sigma-Aldrich) was added to each well at 10% (v/v) and incubated for 2 h 30 min at 37 °C and 5% (v/v) CO_2_, protected from the light. The supernatant was then collected, transferred to a dark 96-well plate (Falcon) and the reduction of resazurin (non-fluorescent blue dye) into resorufin (pink fluorescent) was measured at an excitation wavelength of 530 nm and an emission wavelength of 590 nm, using the spectrophotometer microplate reader Synergy MX (Biotek Synergy). Seven biological replicates were performed for each condition and time-point.

### Cell proliferation assay through Ki-67 immunostaining

MSCs were seeded at 15 000 cells/cm^2^ in 96-well plates, cultured for 3 days in MSC growth media, fixed and immunostained for Ki-67 (1:100; SP6, Thermo Fisher Scientific) followed by Alexa Fluor 647-conjugated secondary antibody (1:1 000; Thermo Fisher Scientific). Nuclei were stained with 1 μg/mL DAPI, for 5 min. The respective manufacturers and dilutions are detailed in Additional file 1: Table SIV. Cells incubated with the secondary antibody alone were used as a negative control. Five biological replicates were performed per condition. Images were acquired in a IN Cell Analyzer 2 000 (GE Healthcare) using a Nikon 20x/0.45 NA Plan Fluor objective and then analyzed using the IN Cell Developer software (GE Healthcare). The total number of nuclei per image was counted, and the Ki-67 positive cells were quantified. Results are presented as the percentage of Ki-67-positive cells per total number of cells (DAPI-positive).

### Cell death/apoptosis assay by flow cytometry and the colorimetric lactate dehydrogenase (LDH) assay

Transfected MSCs were cultured for 2 days before labeling with the FITC-Annexin V Apoptosis Detection Kit I (BD Biosciences), according to manufacturer's instructions. Briefly, approximately 1 × 10^5^ cells were resuspended in binding buffer 1 × and incubated with FITC-Annexin V and Propidium Iodide (PI) for 15 min, protected from the light at RT. Unstained and single cells stained with FITC-Annexin V or PI were used as controls. Flow cytometry was performed within 1 h on the FACS Canto II ™ system (BD Biosciences) and the results were analyzed using the FlowJo software (BD Biosciences). The LDH was measured using the CytoTox 96® Non-Radioactive Cytotoxicity Assay (Promega) and the details are provided in the Additional file 1: Methods section.

### RNA fractionation

Approximately 10 × 10^6^ U2OS cells were collected through trypsinization and washed twice in cold PBS 1x. The cell pellet was resuspended in 1 mL of lysis buffer [10 mM Tris–HCl (pH 8–8.4) 0.14 M NaCl (Merck), 1.5 mM MgCl_2_ (Sigma-Aldrich) and 0.5% (v/v) NP-40 (Sigma-Aldrich)], before collection of 100 μL of the resulting lysates to be labeled as the whole-cell RNA fraction. The nuclei were spun down at 1 000 g for 4 min. The supernatant, representing the cytoplasmic fraction, was collected and centrifuged at 11 000 g for 1 min to remove the remaining nuclei. Nuclei pellets were resuspended in 1 mL of lysis buffer and 1/10 volume (100 μL) of a detergent solution [3.3% (w/v) sodium deoxycholate (Sigma-Aldrich) and 6.6% (v/v) Tween 40 (Merck)] under slow vortexing. The stripped nuclei were then centrifuged at 1 000 g, for 4 min at 4 °C. The nuclei pellet was then washed once more in lysis buffer and spun down at 1 000 g, for 4 min at 4 °C. The pellet enriched in nucleic content was resuspended in 1 mL of TRIzol using a 21-gauge syringe. TRIzol was also added to the whole-cell RNA and cytoplasmatic fraction and RNA was extracted according to the manufacturer’s instructions.

### Decellularization of MSC-derived ECM following osteogenic-inducing stimuli

siH19-MSC or respective control-MSC were cultured for 10 days under osteogenic-inducing conditions to synthesize ECM. The decellularization process was performed in a flow chamber and 1% (v/v) P/S and 0.5% (v/v) fungizone (Capricorn) were added to each solution. The cell culture media was gently removed, cells were washed with PBS 1 × and treated with 0.2% (v/v) Triton X-100 for 10 min at RT. The solution was carefully removed and a 2 M urea (Sigma-Aldrich)/0.2% (v/v) Triton X-100 solution was slowly added, to avoid disturbance of the matrix, and left to incubate for 10 min with gently agitation. The decellularized matrices were treated with 120 Ku/mL of DNase-I (PanReac AppliChem) for 30 min, at 37 °C, and the insoluble ECM layer was washed three times with de-ionized H_2_O to ensure complete removal of the solubilized material.

Decellularization was confirmed by DAPI (1 μg/mL, for 5 min), hematoxylin and eosin staining and by DNA quantification using Pico-Green dsDNA assay (Quant-iT ™ Pico-Green™ dsDNA assay kit, Thermo Fisher Scientific). Non-decellularized matrices were used as control. Further details are provided in the Additional file 1: Methods section.

### Quantification of glycosaminoglycans (GAGS) and collagen content

Content of GAGs in the decellularized matrices was measured using Blyscan™ GAG assay (Biocolor), according to the manufacturer’s instructions (Additional file 1: Methods). The amount of collagenous proteins in the decellularized matrices was measured using the SIRCOL kit (Biocolor), according to the manufacturer’s instructions (Additional file 1: Methods).

### Atomic force microscopy (AFM)

AFM topography images of the ECM were obtained with a PicoPlus scanning probe microscope interfaced with a Picoscan 5500 controller (Keysight Technologies) using the PicoView 1.20 software (Keysight Technologies), coupled to an Inverted Optical Microscope (Observer Z1, Zeiss). Each sample was imaged with a 100 × 100 µm [2] piezoelectric scanner. All measurements were performed in contact mode, in air, at RT, using bar-shaped cantilever silicon tips (MLCT-Bio-DC, Bruker) with a spring constant of 0.03 N/m. The scan speed was set at 2.0 Hz.

### Recellularization of the siH19-engineered matrices

Decellularized matrices were incubated with cell culture media supplemented with 1% (v/v) P/S and 0.5% (v/v) fungizone at 37 °C in sterile conditions, for 4 h. Naïve MSCs or pre-adipocytes (differentiated from MSCs cultured for 10 days in adipogenesis-inducing conditions) were detached with trypsin–EDTA, centrifuged, resuspended in fresh media with 1% (v/v) P/S and 0.5% (v/v) fungizone, and plated on top of the decellularized sheets at a cell density of 10 000 cells/cm^2^.

### Histology and histomorphometry analysis in bone samples

Bone samples were divided for histologic and histomorphometric evaluation. Samples were cut and fixed in 10% (w/v) formalin (Bio-Optica) for 3 days. For histomorphometric analysis, undecalcified samples were processed for plastic embedding, using a previously described protocol. Briefly, samples were dehydrated in a graded series of ethanol [50, 70 and 100% (v/v)], followed by immersion in xylol for 24 h. The undecalcified bone samples were infiltrated with a plastic embedding mixture using a three-step protocol. In each step, the samples were infiltrated for three consecutive days with daily freshly made solutions, containing 75% (v/v) of methylmethacrylate (MMA, Sigma-Aldrich) and 25% (v/v) of dibutyl phthalate (Prolabo) with increase in concentrations (0 g/mL; 0.01 g/mL and 0.025 g/mL) of benzoyl peroxide (Sigma-Aldrich). Polymerization was carried out at 37 °C for a week. The plastic blocks, containing the processed bone samples, were cut into 7 µm sections using a tungsten knife (Leica). After deplasticization, the sections were stained with toluidine blue staining. Trabecular separation (Tb.Sp) was determined using the Osteomeasure bone histomorphometry software (OsteoMetrics, OsteoMetrics, Inc.). The percentage of adipose tissue was calculated using a 15 to 11 points grid [38, 39].

### Statistical analysis

Statistical data analysis was performed using GraphPad Prism 7 (GraphPad Software, Inc.). Gaussian distribution was tested by the Shapiro–Wilk and Kolmogorov–Smirnov tests. For data with a normal distribution, parametric tests were used to evaluate differences between samples, namely a Student *t* test (between two groups) or one-way ANOVA (more than two groups). For non-normalized data, Mann–Whitney U test (between two groups) or Kruskal–Wallis, followed by Dunn’s multiple comparisons test (more than two groups) were used. Categorical data was tested using the Fisher's exact test. Pearson or Spearman correlation coefficients were calculated when the data followed a parametric and nonparametric distribution, respectively. Statistical significance was considered for *p* < 0.05 (* *p* < 0.05; ** *p* < 0.01 and *** *p* < 0.001).

## Results

### *H19* controls matrisome of MSC-derived pre-osteoblasts

**Fig. 1:**
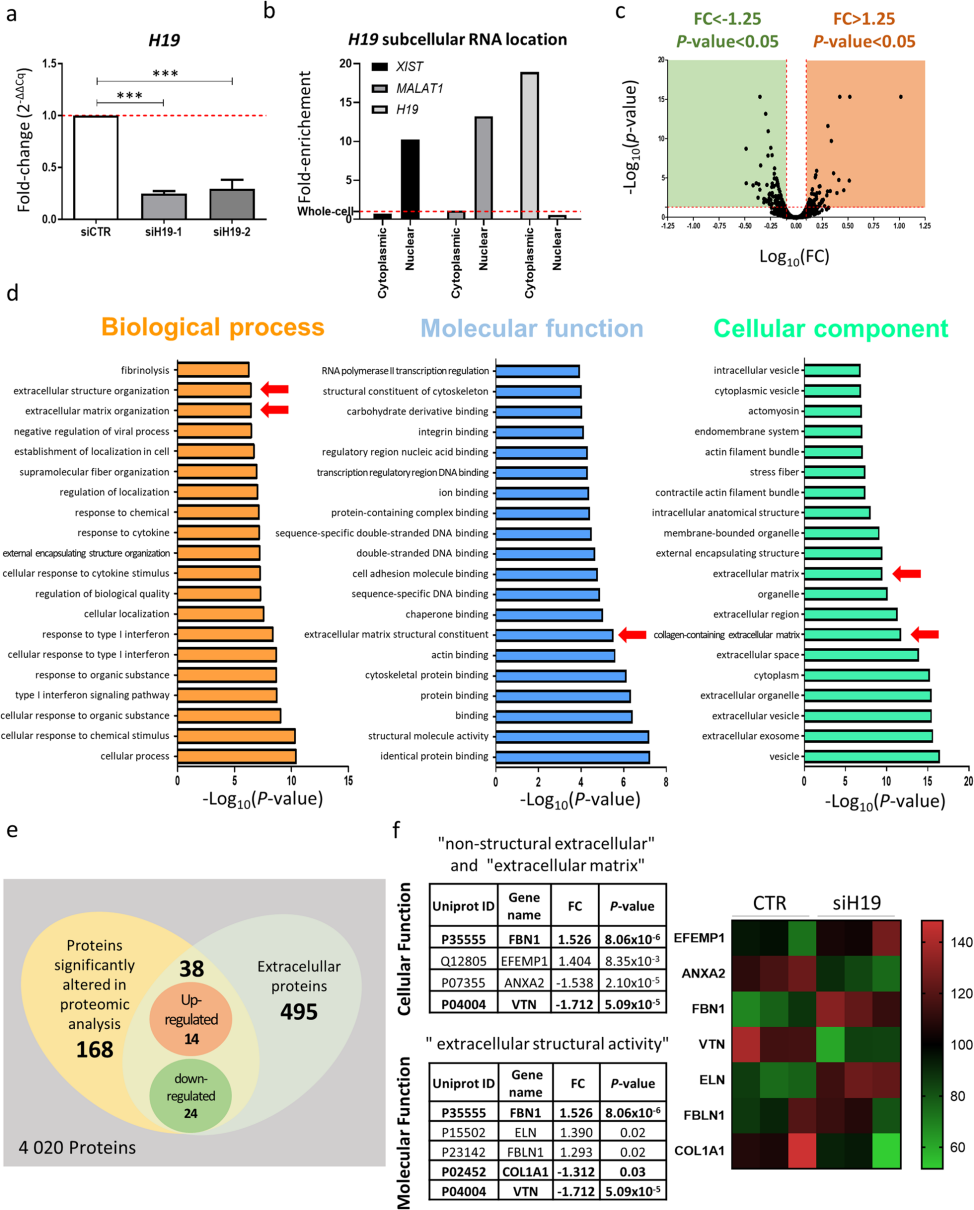
Proteomic analysis of *H19* downstream targets and ECM-related processes. **a** *H19* expression after transfection of MSCs, isolated from osteoporotic patients, with two alternative small interference RNAs against *H19* (siH19-1 and siH19-2) or the respective control (siCTR), cultured for 3 days in osteogenic-inducing conditions (*N* = 6). **b** *H19* subcellular location in U2OS osteosarcoma cells. Levels were normalized against the whole-cell content. *XIST* and *MALAT1*, two nuclear lncRNAs, were used as quality controls. **c** Volcano plot of the proteins identified after transfection with siH19 versus control (FC ≥ |1.25|) and *p*-value < 0.05. The y-axis corresponds to the − Log_10_(*p*-value) of a certain protein and the x-axis the correspondent Log_10_(FC). **d** Gene ontology (GO) enrichment analysis, according to PANTHER classification system. The x-axis shows the −Log_10_(*p*-value) associated with determined GO term (y-axis). **e** Venn diagram showing the extracellular proteins differentially expressed by *H19* knockdown. **f** Proteins associated with the GO terms “non-structural extracellular” and “extracellular matrix” with *p* < 0.01 and a minimum of four unique peptides (left) and with the GO term “extracellular structural activity” (right) and respective heatmap.

To screen for matrisome-specific proteins regulated by *H19*, we performed a mass spectrometry-based quantitative proteomic analysis in MSCs isolated from osteoporotic patients, following induction into the osteogenic lineage. In these cells, the levels of *H19* were successfully reduced by transfection with siH19, with an average efficiency of  > 75% and 70% decrease for siH19-1 and siH19-2, respectively (Fig. 1a). Thus, siH19-1 was the sequence selected for the following experiments. Cell fractionation shows that, contrary to other typically nuclear lncRNAs, such as *XIST* and *MALAT1*, *H19* transcripts are mainly located in the cytoplasm (Fig. 1b). A matrix enrichment buffer was used to facilitate the solubilization of ECM proteins derived from siH19-MSCs and siCTR-MSCs. Proteomic profiling identified 4 020 proteins, considering a minimum of two unique peptides. Results show that 66 proteins were significantly upregulated [Fold-change (FC) > 1.25; *p* < 0.05], whereas 102 were significantly downregulated (FC < -1.25; *p* < 0.05), in MSCs with reduced levels of *H19* compared with the control (Fig. 1c).

The GO analysis on the biological processes revealed that extracellular matrix organization (GO:0030198; fold-enrichment (FE) = 5.55, *p* = 3.52 × 10^–7^; false discovery rate (FDR) = 3.09 × 10^–4^) and extracellular structure organization (GO:0043062; FE = 5.54, *p* = 3.63 × 10^–7^; FDR = 3.01 × 10^–4^) are among the top 20 terms with higher significance and low FDR (Fig. 1d). Further analysis revealed that the extracellular matrix structural constituent (GO:0005201) is the fourth term with the highest FE score, and is within the top ten with the lowest *p-*value (*p* = 2.98 × 10^–6^; FDR = 2.03 × 10^–3^) (Fig. 1d). The following proteins are associated with this term: collagen alpha-1(I) chain (COL1A1; P02452); EGF-containing fibulin-like extracellular matrix protein 1 (EFEMP1; Q12805); collagen triple helix repeat containing 1 (CTHRC1; Q96CG8); fibrinogen beta chain (FGB; P02675); fibrillin-1 (FBN1; P35555); fibulin-1 (FBLN1; P23142); elastin (ELN; P15502); vitronectin (VTN; P04004) and lactadherin (MFGE8; Q08431). Among the 20 most enriched cellular components terms are collagen-containing extracellular matrix (GO:0062023; FE = 7.45, *p* = 1.67 × 10^–12^; FDR = 4.75 × 10^–10^) and extracellular matrix (GO:0031012; FE = 5.56, *p* = 3.09 × 10^–10^; FDR = 6.16 × 10^–8^) (Fig. 1d). Also, IPA shows that the growth hormone signaling pathway and the Wnt/β-catenin signaling pathway are predicted to be changed by *H19* knockdown.

A Venn diagram was used to compare the differently expressed proteins from the proteomic analysis and the GO cellular terms associated with ECM proteins. Results show that 38 of the differently expressed proteins in the siH19-engineered MSCs versus control MSCs are associated with the cellular functions “extracellular matrix” and “non-structural extracellular” (Fig. 1e). Out of those, 14 proteins are upregulated, while 24 are downregulated following *H19* knockdown, compared with the control. Specifically, the differently expressed proteins FBN1 (FC = 1.53, *p* = 8.06 × 10^–6^), VTN (FC = − 1.72, *p* = 5.09 × 10^–5^) and COL1A1 (FC = − 1.31, *p* = 0.03) are associated with ECM-related GO terms (Fig. 1f), while CTHRC1 (FC = − 1.374, *p* = 0.0085) is associated with collagen biogenesis.

### ECM components are regulated by *H19*

**Fig. 2:**
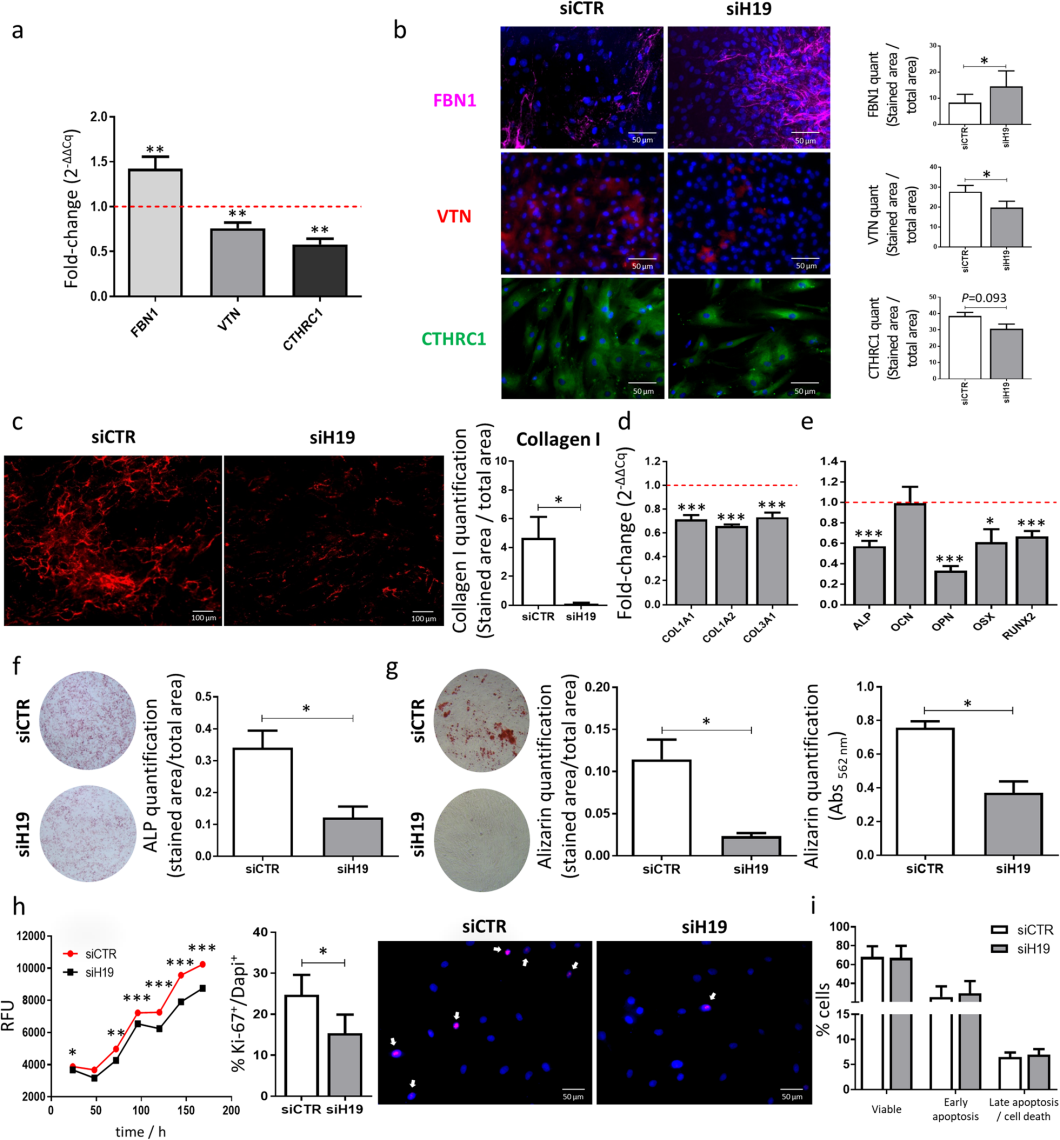
Effect of *H19* knockdown on osteogenic differentiation, ECM composition, proliferation and apoptosis in MSCs. **a** mRNA expression levels of FBN1, VNT and CTHRC1 after *H19* knockdown compared with control (*N* = 6). **b** Representative image and quantification of FBN1, VTN and CTHRC1 protein levels 10 days after culture in osteogenic-inducing conditions (*N* = 6; scale: 50 μm). **c** COL1 staining (left) and quantification (right) at day 7 of differentiation (COL1: red; *N* = 6; scale: 100 μm). **d** ECM components (*COL1A1*, *COL1A2* and *COL3A1*) and **e** Osteogenic markers (*ALP* and *RUNX2*) expression levels at day 3 after transfection and incubation in osteogenic-inducing conditions (*N* = 6). **f** ALP (*N* = 6) and **g** Alizarin (*N* = 6) staining after transfection and culture for 7 and 14 days, respectively, in osteogenic-inducing conditions. **h** Representative fluorescence profile (resorufin) measured every 24 h for 7 days in transfected MSCs; quantification of the percentage of cells in proliferation (ki-67^+^/DAPI^+^) 3 days after siH19 or siCTR transfection; and representative images of MSC stained for the proliferation marker Ki-67 protein (red) and nuclear DNA labeled with DAPI (blue) (*N* = 6; scale: 50 μm) (RFU: relative fluorescence units). **i** Flow cytometry analysis of Annexin V/PI staining and quantification of viable (Annexin V^−^ PI^−^), early apoptotic (Annexin V^+^ PI^−^), or late apoptosis and dead cells (Annexin V^−^ PI^+^ or Annexin V^+^ PI^+^) (*N* = 6). siCTR: MSC transfected with scrambled control sequence; siH19: MSC transfected with small interference RNA against *H19*.

To validate novel ECM proteins regulated by *H19*, the levels of *FBN1*, *VTN* and *CTHRC1* were evaluated at the transcriptional level. RT-qPCR results show a 42.5% upregulation for *FBN1* and a 24.1% and 42.0% downregulation for *VTN* and *CTHRC1*, respectively, in siH19-MSCs compared with the control, following 7 days of osteogenic differentiation (Fig. 2a). Furthermore, immunocytochemistry was used to detect these proteins in the MSC-derived matrices. In agreement with the RT-qPCR, results show an increase for FBN1, while VTN is decreased in siH19-MSCs versus control MSCs (Fig. 2b). Although not statistically significant, these also show a tendency for a reduction in CTHRC1 protein levels (Fig. 2b).

Being collagen type I (COL1) a major component of bone ECM, its levels were also quantified by immunocytochemistry. COL1 is reduced in siH19-MSCs versus control MSCs (97.4%), following 7 days of osteogenic induction (Fig. 2c). In agreement, mRNA expression of *COL1A1*, *COL1A2* and *COL3A1* is reduced by siH19 (Fig. 2d)*.* The same decrease in the levels of these transcripts is also present when using a distinct sequence for *H19* silencing (siH19-2; Additional file 1: Fig. S1).

### *H19* impairs osteogenic differentiation and mineralization

To evaluate the biological impact of *H19* on MSCs, loss-of-function studies were performed. The decrease in *H19* levels caused a reduction in expression levels of *ALP*, *OPN*, *OSX* and *RUNX2*, four early osteogenic markers (Fig. 2e), but did not change the expression of the late osteogenic marker *OCN*. This was further confirmed through ALP staining, which shows a significant decrease (*p* < 0.05) in siH19-MSCs compared with the control, after culture for 7 days in osteogenic-inducing conditions (Fig. 2f). Furthermore, *H19* knockdown revealed a 79.7% decrease (*p* < 0.05) in the deposition of calcium nodules after 10 days of differentiation (Fig. 2g), which demonstrates that *H19* has an impact on mineralization.

Moreover, results also show a significant reduction in MSCs proliferation, assessed through measurement of the metabolic activity, after *H19* depletion compared with control MSCs (Fig. 2h). To further confirm this, cells were stained for the proliferation marker Ki-67. Results show that *H19* disturbs the MSCs proliferative capacity, considering the reduction in the percentage of Ki-67^+^/DAPI^+^ (*p* < 0.05) in the siH19-MSCs (Fig. 2h). Nevertheless, flow cytometry shows that the inhibition of *H19* does not affect early apoptosis or late apoptosis/cell death (Fig. 2i). Taken together, these results indicate that the inhibition of *H19* expression exerted an osteogenic- and proliferative-suppressive effect in MSCs in vitro.

### ECM derived from siH19-engineered cells affects the behavior of naïve MSCs

**Fig. 3:**
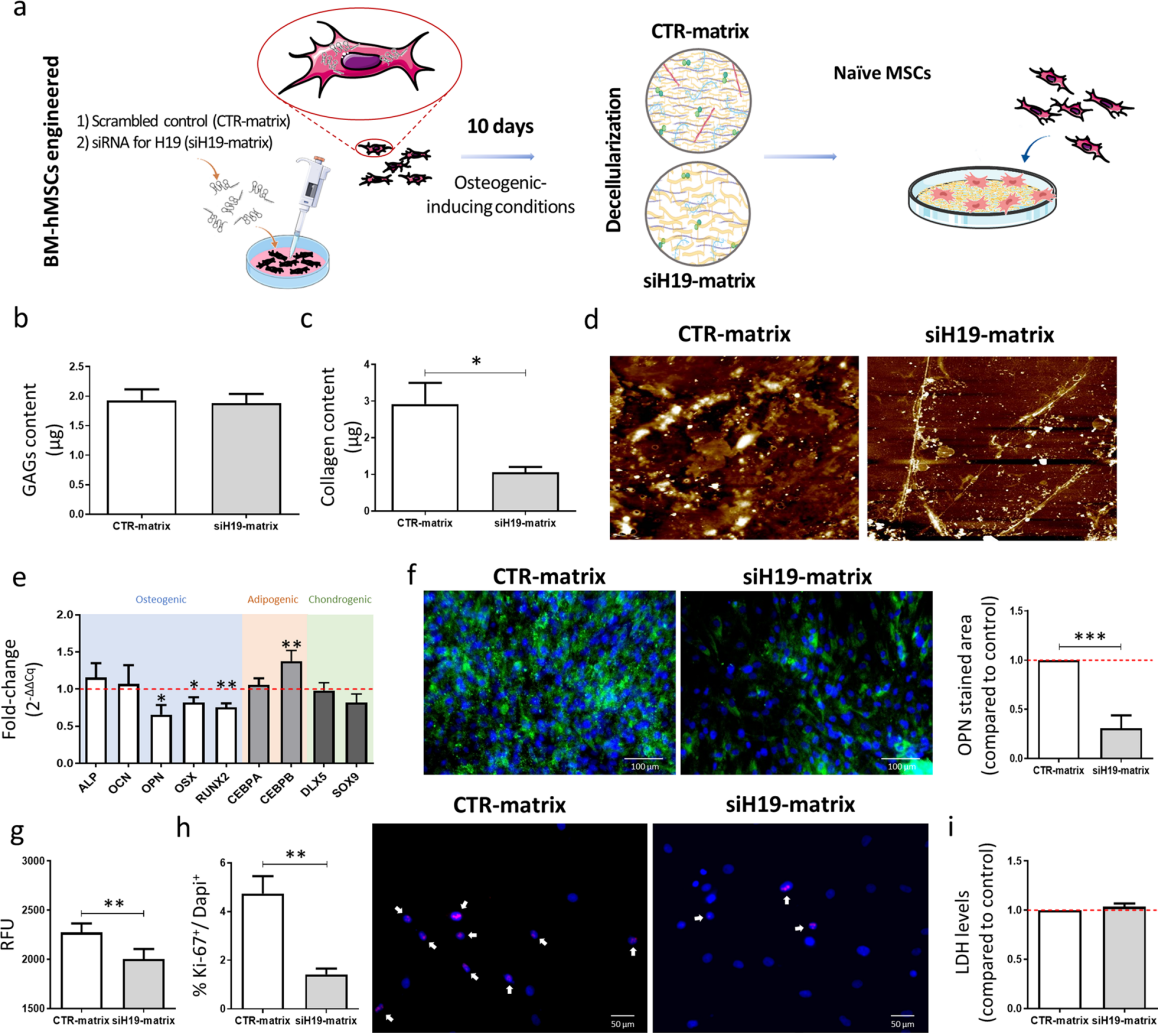
Decellularized ECM produced by siH19-engineered MSC and its effect on naïve MSC. **a** Schematic representation of the experiment. **b** sGAGs and **c** collagenous content quantification after decellularization of the matrices produced by control-MSC (CTR-matrix) or siH19-MSC (siH19-matrix) cultured under osteogenic-inducing conditions (*N* = 6). **d** Representative AFM image of the topography of matrices produced by MSC depleted in *H19* and control, after 10 days in osteogenic-inducing conditions, and following the decellularization process. **e** Expression levels of osteogenic, adipogenic and chondrogenic markers assessed by RT-qPCR, 7 days after repopulation of decellularized matrices with naïve MSCs (*N* = 6). **f** Representative image and quantification of immunostaining of Osteopontin (OPN: green; Nuclei: blue) (*N* = 6; scale: 100 μm). **g** Measurement of the metabolic activity through resazurin assay (*N* = 6; scale: 50 μm). **h** Representative image of MSC stained for the proliferation marker Ki-67 protein (red) and nuclear DNA labeled with DAPI (blue); and quantification of the percentage of MSC in proliferation (Ki-67^+^/DAPI^+^) (*N* = 6) after 7 days of culture on top of decellularized matrices. **i** Quantification of LDH levels on the conditioned-media from naïve MSC cultured on the decellularized matrices (*N* = 6).

Considering the impact of *H19* on the ECM components and osteogenesis, we next evaluated the osteogenic behavior of naïve MSCs on the matrices derived from siH19 cells (Fig. 3a). Firstly, MSCs were transfected with either siH19 or the control, and were allowed to differentiate for 10 days in osteogenic-inducing conditions to promote the deposition of an ECM network. Next, matrices were decellularized and characterized. No DAPI-positive cells or Gill’s hematoxylin stained cells were found in the decellularized matrices, compared with non-decellularized MSCs, which is in agreement with the quantification of low amounts of DNA content (Additional file 1: Fig. S2). There are no differences in GAGs content, when comparing decellularized matrices derived from siH19-MSCs with control MSCs (Fig. 3b). As expected, the collagenous content in decellularized matrices derived from siH19-MSCs, following osteogenic differentiation, is significantly reduced (63.8%, *p* < 0.05) compared with the controls (Fig. 3c). Furthermore, AFM microscopy images of the decellularized matrices show the presence of a continuous and thicker layer of ECM on the control matrix, in opposition to a thinner and porous network of extracellular matrix fibers on the siH19-derived matrices (Fig. 3d), suggesting an impairment in the ECM production by *H19* silencing.

To evaluate if the changes in the matrices secreted by siH19-engineered MSCs influence MSC lineage commitment, naïve cells were used to repopulate the decellularized matrices. Following 7 days of culture, results show that *OPN*, *OSX* and *RUNX2*, three osteogenic key markers, are significantly downregulated, while *CEBPB,* an adipogenic marker is significantly upregulated, when MSCs are cultured on top of siH19-derived matrices, compared with control-derived matrices (Fig. 3e). No significant differences in gene expression were found for *ALP*, *OCN* and *CEBPA*, nor for the chondrogenic markers, *DLX5* and *SOX9* (Fig. 3e). Furthermore, immunocytochemistry results show that OPN protein is decreased when naïve MSCs are reseeded on siH19-derived matrices (Fig. 3f), suggesting that the secreted matrices are detrimental for osteogenesis when *H19* levels are reduced.

To evaluate if the MSC proliferation is influenced by siH19-derived matrices, naïve MSCs were cultured on top of the decellularized matrices. Results show a decrease in the metabolic activity (Fig. 3g) and in the proliferative capacity (Fig. 3h), evaluated by the percentage of Ki-67^+^/DAPI^+^ cells, in naïve MSC cultured on top of siH19-decellularized versus control-decellularized matrices. Also, levels of LDH on the conditioned-media were measured to assess cell viability/cytotoxicity. No differences were found between MSCs cultured on top of the different matrices (Fig. 3i), with LDH levels being similar to those detected when cells were cultured in tissue culture treated plastics (TCPS) flasks, which excludes a cytotoxic effect. Overall, these results indicate that changes in the matrix induced by siH19 affect the lineage commitment and the proliferative capacity of naïve MSCs derived from osteoporotic patients.

### ECM derived from siH19-engineered cells affect the behavior of pre-adipocytes

**Fig. 4:**
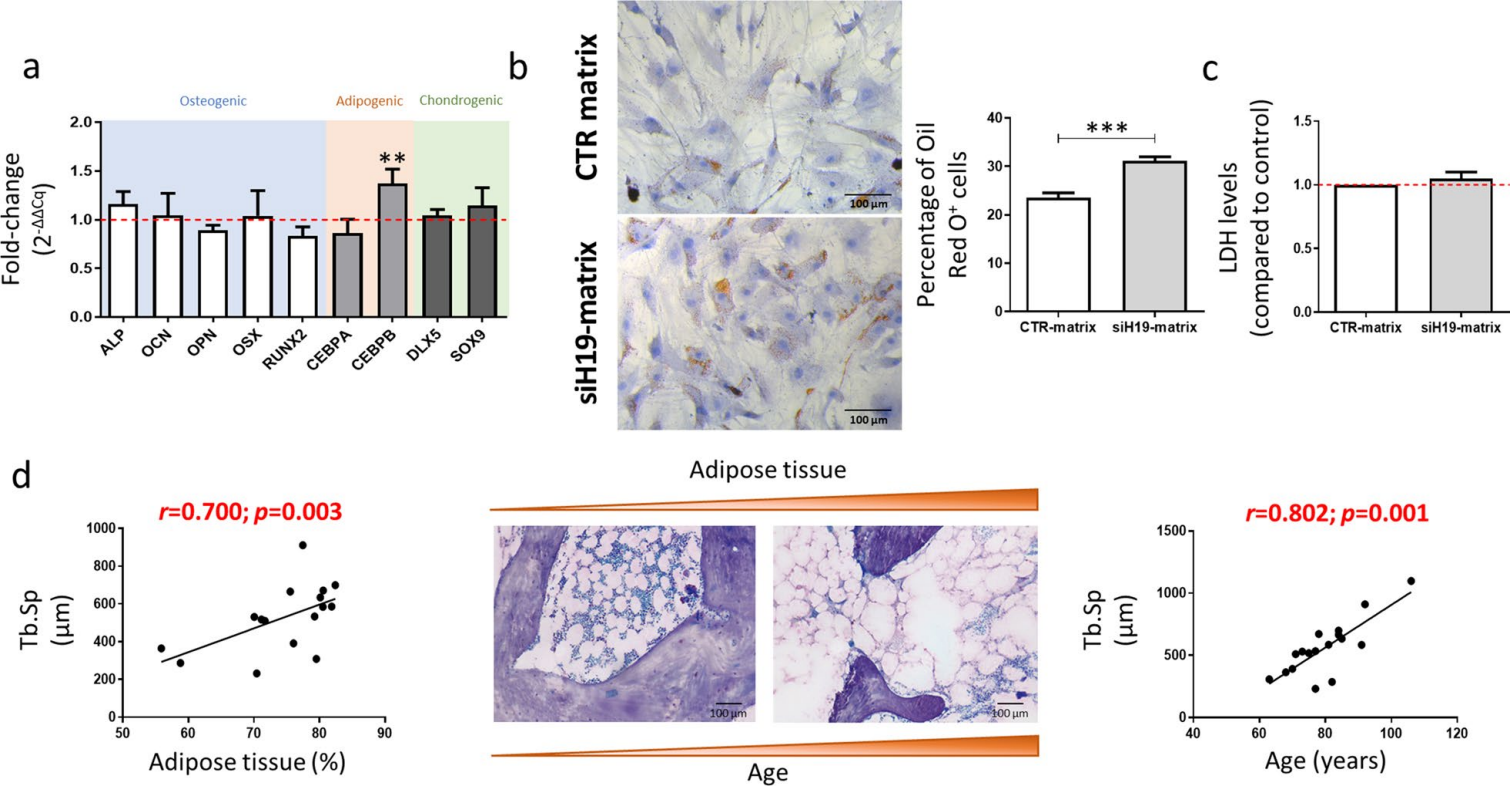
Effect of siH19-MSCs derived matrices on pre-adipocytes phenotype. **a** Osteogenic, adipogenic and chondrogenic markers expression levels assessed by RT-qPCR, 7 days after repopulation with pre-adipocytes on decellularized matrices (*N* = 6). **b** Representative image and quantification of the percentage of positive cells for the Oil Red O staining after 3 days of culture on decellularized matrices (Hematoxylin: blue-purple color; Oil Red O^+^ staining: red color) (*N* = 6; scale: 100 μm). **c** Quantification of LDH levels on the conditioned-media of pre-adipocytes cultured on decellularized matrices (*N* = 6). **d** Correlation of trabecular separation (Tb.Sp) with the percentage of adipose tissue in bone samples from patients with fragility fractures (*N* = 16) (left panel). Representative images (scale: 100 μm) and correlation coefficient of trabecular separation (Tb.Sp) and age (*N* = 17) (right panel).

The commitment of MSC into the osteogenic lineage instead of the adipogenic lineage is crucial for a healthy bone. To understand the impact of the matrices on the behavior of pre-adipocytes, MSCs were differentiated for 10 days in adipogenic-inducing conditions (pre-adipocytes), and then used to reseed the decellularized matrix produced by osteogenic-induced MSCs with *H19* reduced levels or control. Results show that after 7 days in culture, the expression of *CEBPB* was significantly increased (*p* < 0.01) on pre-adipocytes cultured on top of the siH19-derived matrix, compared with cells cultured on top of the control matrix, with no differences regarding osteogenic or chondrogenic markers (*ALP*, *OCN*, *OPN*, *OSX* and *RUNX2*; *DLX5* and *SOX9*) (Fig. 4a). Interestingly, changes in the number of cells with lipid droplets (stained with Oil Red O) were observed with only 3 days of culture on top of the decellularized matrices. Specifically, the percentage of Oil Red O^+^ cells was significantly increased in the siH19-derived matrices (*p* < 0.001) (Fig. 4b), compared with the control matrices, following normalization to the total number of cells (number of nuclei stained with hematoxylin). Similarly to the results from naïve MSC, we did not detect a cytotoxic effect of the decellularized matrices on the pre-adipocytes (Fig. 4c). Globally, the results suggest that *H19* knockdown promotes adipogenesis and increases the number of cells with lipid droplets. Interestingly, in bone clinical samples from osteoporosis patients, we found a significant positive correlation between the percentage of area covered by adipose tissue and the trabecular separation (Tb.Sp), which also positively correlates with age (Fig. 4d).

### miR-29c-3p controls *H19* expression

**Fig. 5:**
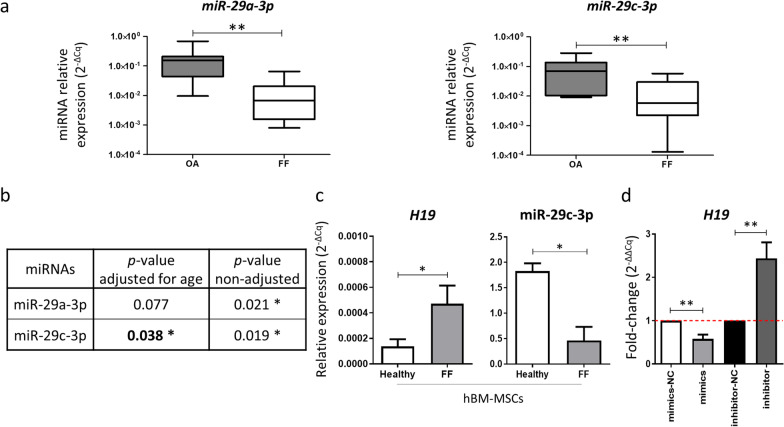
miR-29c-3p expression in bone samples and the impact of miR-29c-3p in *H19* levels. **a** Box and whiskers plots show miRNA expression levels (2^−ΔCq^) in bone samples from patients with fragility fractures (FF) or osteoarthritis (OA). **b** Adjusted *p*-values for miRNA expression considering age. **c** Expression levels of *H19* and miR-29c-3p on MSC from healthy and FF patients (*N* = 3). **d** Effect of the modulation of the levels of miR-29c-3p on the lncRNA *H19* expression, 3 days post-transfection in osteogenic-inducing conditions (*N* = 6). (Mimics-NC: MSC transfected with scrambled miRNA mimics negative control; Mimics: miR-29c-3p transfected MSC; Inhibitor-NC: MSC transfected with scrambled miRNA inhibitor negative control; Inhibitor: miR-29c-3p-inhibitor transfected MSC)

To identify potential regulators of *H19*, an in silico analysis on miRNAs was performed using the ENCORI platform (http://starbase.sysu.edu.cn/panCancer.php), and potential binding sites for the miR-29 family, including miR-29a, miR-29b and miR-29c members, are shown in Additional file 1: Fig. S3. To understand the relevance of those miRNAs in osteoporosis, we evaluated their expression levels in bone samples from osteoporotic fragility fractures (FF) and osteoarthritis (OA) patients. RT-qPCR results show that miR-29b-3p is poorly expressed in the bone samples or even undetected (C_q_ > 35). However, miR-29a-3p and miR-29c-3p were expressed and significantly decreased in FF versus OA bone samples (Fig. 5a). A multivariate analysis was performed to exclude possible associations between miRNA expression and clinically relevant variables, including age. When using miRNA expression and age as independent variables, and disease as a dependent variable, the results from the multivariate linear regression analysis with the stepwise forward method show that only miR-29c-3p, but not miR-29a-3p, is statistically significant (Fig. 5b), being a strong predictive factor of the disease. The levels of miR-29c-3p were also decreased in MSC isolated from osteoporosis patients compared with healthy donors. In the same samples, *H19* expression shows an antagonistic profile, being upregulated in MSC derived from osteoporosis patients versus healthy donors (Fig. 5c). Interestingly, during the early stages of osteogenic differentiation *H19* expression is increased in MSC from healthy donors (*p* < 0.05, day 7). However, this increase is not present during osteogenic differentiation of osteoporosis-derived MSC, even in the presence of osteogenic-inducing stimuli (Additional file 1: Fig. S4). This reinforces the need for studies to be conducted in samples derived from osteoporosis patients.

Next, to evaluate if miR-29c-3p regulates *H19*, the expression levels of *H19* were tested after modulation of miR-29c-3p in MSCs cultured for 3 days in osteogenic-inducing conditions (Additional file 1: Fig. S5). Results show that miR-29c-3p overexpression significantly decreases *H19* (FC = 0.58), while miR-29c-3p inhibition increases *H19* levels (FC = 2.44). (Fig. 5d).

### miR-29c-3p reduces collagen expression and MSC proliferation but do not impact ALP and mineralization

**Fig. 6:**
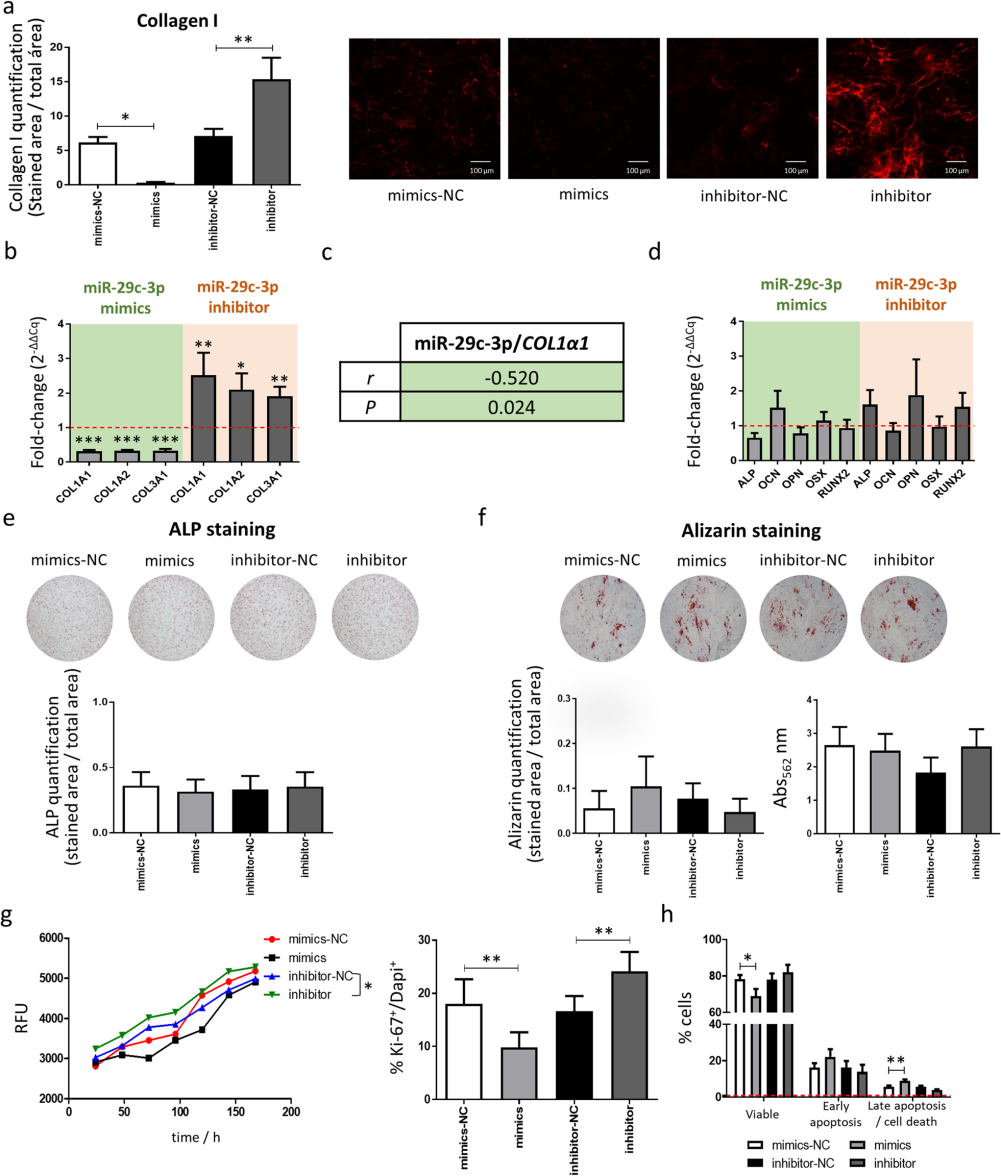
Effect of miR-29c-3p in ECM composition, osteogenic differentiation, cell proliferation and apoptosis. **a** COL1 staining and quantification at day 7 of differentiation. (COL1: red; *N* = 6; scale: 100 μm) **b** Expression of ECM components (*COL1A1*, *COL1A2* and *COL3A1*) by qPCR after 3 days in osteogenic-inducing conditions (*N* = 6). **c** Correlation between miR-29c-3p expression level and collagen expression on bone samples. **d** *E*xpression levels of osteogenic markers at day 3 after transfection and incubation in osteogenic-inducing conditions (*N* = 6). **e** ALP and **f** Alizarin staining (*N* = 6) and Alizarin solubilization quantification (*N* = 6), after transfection and culture for 7 and 14 days, respectively, in osteogenic-inducing conditions. **g** Representative fluorescence profile (resorufin) measured every 24 h for 7 days in transfected MSCs and quantification of the percentage of cells in proliferation (Ki-67^+^/DAPI^+^) after 3 days in culture (*N* = 6). **h** Quantification of viable (Annexin V^−^ PI^−^), early apoptotic (Annexin V^+^ PI^−^), or late apoptosis and dead MSCs (Annexin V^−^ PI^+^ and Annexin V^+^ PI^+^) (*N* = 6). (Mimics-NC: MSC transfected with scrambled miRNA mimics negative control; Mimics: miR-29c-3p transfected MSC; Inhibitor-NC: MSC transfected with scrambled miRNA inhibitor negative control; Inhibitor: miR-29c-3p-inhibitor transfected MSC).

To understand if miR-29c-3p impacts *H19*-mediated processes, collagen secretion, ALP level, mineralization, proliferation and apoptosis in MSCs were investigated. Immunostaining against COL1, after 7 days of culture in osteogenic-inducing conditions, shows that COL1 proteins are reduced by 94.4% when MSCs are transfected with miR-29c-3p mimics, while COL1 protein levels are highly increased following inhibition of miR-29c-3p, compared with the respective controls (Fig. 6a). At the transcription level, a reduction was found for *COL1A1*, *COL1A2* and *COL3A1* in miR-29c-3p-overexpressing-MSCs, whereas the opposite was observed in miR-29c-3p-depleted-MSCs (Fig. 6b). Interestingly, in osteoporotic bone samples, there is a negative correlation between miR-29c-3p expression levels and *COL1A1* (Fig. 6c). To test if miR-29c-3p regulates osteogenesis, osteogenic differentiation markers, ALP staining and mineralization were analyzed. However, no differences were found, suggesting that the modulation of miR-29c-3p does not impair osteogenic differentiation, ALP or calcium deposition (Fig. 6d, 6e and 6f). On the other hand, differences were found regarding proliferation. The metabolic activity is significantly increased when miR-29c-3p levels are reduced and, although not statistically significant, there is a tendency for a reduction in the metabolic activity following miR-29c-3p overexpression (Fig. 6g). These results were confirmed when analyzing the proliferation marker Ki-67. The percentage of Ki-67^+^/DAPI^+^ is significantly decreased by miR-29c-3p and increased following miR-29c-3p inhibition (Fig. 6g). Furthermore, analysis of Annexin V/PI staining by flow cytometry showed that the miR-29c-3p overexpression significantly decreases the percentage of viable cells, while increasing the percentage of cells in late apoptosis/cell death (Fig. 6h). Taken together, these results suggest that miR-29c-3p regulates levels of collagen proteins and MSCs proliferation, but do not impair ALP or mineralization.

## Discussion

In osteoporosis, the composition and structure of the organic and inorganic bone ECM are affected by the disruption in the interplay between osteoblasts and osteoclasts [11, 19]. In order to restore bone ECM, it is essential to identify the key parameters that regulate ECM components. On one hand, ECM proteins are regulated by RNA transcripts; on the other hand, the ECM is able to modulate the transcription of certain RNAs. LncRNAs have been gaining interest as therapeutic targets for bone diseases, due to their involvement in bone metabolism, remodeling and metastasis [6]. However, up-to-date, the role of lncRNAs has only been revealed for a small portion of transcripts.

LncRNA *H19* is a pro-osteogenic molecule that has been found to be deregulated in osteoporosis, both in human patients [40, 41] and animal models [34, 41, 42]. In this study, we performed, for the first time, an ECM-enriched proteomic analysis in *H19* depleted MSCs derived from osteoporotic patients, to screen for novel matrix proteins regulated by *H19*. Besides collagen synthesis, our study reveals that *H19* modulates multiple ECM proteins, including FBN1, VTN and CTHRC1, among others. Specifically, FBN1 is a structural glycoprotein, that together with fibrillin 2 (FBN2), composes 1–3% of the skeleton [43]. Both proteins regulate the bioavailability of locally produced transforming growth factor beta (TGF-β) and bone morphogenetic protein (BMP) complexes, affecting osteoblast maturation [44–46]. Mutations in FBN1 result in skeletal abnormalities [47]. In contrast, the ECM glycoprotein VTN is a key positive regulator of the mammalian tissue repair and remodeling activity [48–50]. Recently, a vitronectin-derived peptide was shown to reverse ovariectomy-induced bone loss due to its dual antagonist role on bone formation and resorption [51]. Moreover, it mediates cell attachment, spreading and migration to the ECM [51, 52], and was identified as a survival factor for MSCs submitted to serum deprivation-induced apoptosis [53], highlighting its importance as a strategy to increase MSCs survival post-transplantation. Furthermore, the adhesion of MSC to VTN promotes osteogenic differentiation by increasing OCN, COL1 and ALP levels [54]. It also induces mineralization in the presence or absence of osteogenic-inducing supplements. Considering this, the decrease in VTN caused by *H19* depletion may partly explain the decrease in the osteogenic capacity of MSCs, when reseeded on top of the siH19-decellularized matrices. Futhermore, transcript levels of the glycoprotein CTHRC1 were reduced by *H19* silencing. This protein has been widely described to promote cell migration [55] and abundant expression levels of *CTHRC1* are detected in the bone matrix during embryogenesis and in adult mice [56]. Ablation of CTHRC1 results in the impairment of bone formation [57], while increased levels of CTHRC1 stimulate differentiation and mineralization of osteoprogenitor cells [57, 58].

The present study demonstrates that *H19* is crucial for ECM protein production (including collagen, FBN1, VNT, CTHRC1, among others), MSC differentiation into osteoblasts, mineralization and proliferative capacity. Importantly, lncRNAs not only mediate intracellular molecular interactions but may have an indirect effect on the interactions between the ECM and the cells. As such, we demonstrated that the decellularized matrices produced by *H19*-depleted MSCs, cultured under osteogenic-inducing conditions, are sufficient to shift the MSCs commitment into the adipogenic, in detriment of the osteogenic lineage, and to negatively impact MSCs proliferation. Considering levels of multiple ECM proteins and ECM-associated proteins are modulated by *H19*, this effect is probably the consequence of the simultaneous changes in multiple proteins, rather than individual molecules.

Furthermore, we showed that the *H19* expression is regulated by miR-29c-3p, whose levels are decreased in bone samples from osteoporotic patients, and that it negatively changes COL1 expression, which is in agreement with previous studies [59, 60]. In a recent study published by our group, miR-29c-3p was identified as a marker of secondary osteoporosis in multiple myeloma plasma samples [61]. Modulation of miR-29c-3p affects MSC proliferative capacity, without impacting cell death or osteogenic differentiation and mineralization in the early stages of differentiation. Thus, although miR-29c-3p regulates *H19* expression, the functions of these two transcripts do not fully overlap, since differences were found for *H19* regarding osteogenesis, mineralization and several bone ECM proteins. Importantly, the regulation of *H19* by miRNAs is extended far beyond the miR-29 family, as several other miRNAs have been shown to interact with *H19* [28, 32, 33]. On the other hand, the function of miR-29c-3p is not only determined by the targeting of a single molecule but by the balance in levels of all the regulated transcripts, which might explain why the functions of the lncRNA *H19* and the miR-29c-3p are only partially complementary.

Collectively, the findings presented in this study support *H19* as a potential therapeutic target for osteoporosis and repair of fragility fractures through the modulation of the ECM components and the ECM-cell interactions.

## Conclusions

Matrisome signature is altered following *H19* silencing and among the proteins impacted were FBN1, VTN and CTHRC1. siH19-engineered matrices impair MSC proliferation and fate, while enhancing lipid droplets formation in pre-adipocytes. *H19* and miR-29c-3p are antagonistic partners with partially independent functional profiles.

In summary, this study advances lncRNA-based therapeutics for the control of ECM homeostasis.

## Supplementary Information

**Additional file 1: Fig. S1.** Effect of siH19-2 on the expression levels of collagens, osteogenic markers and other H19-target candidates, assessed through RT-qPCR, 3 days after culture under osteogenic-inducing conditions (*N* = 6). **Fig. S2.** Decellularization of MSCs matrices. Primary MSCs from osteoporotic patients were differentiated for 10 days under osteogenic-inducing conditions before decellularization. After treatment with the decellularization buffer (urea 2 M), samples were stained with a) DAPI (blue) to visualize nuclei (scale: 100 μm) and b) hematoxylin (blue-purple color) to visualize nuclei and matrix integrity (scale: 100 μm). c) DNA and sGAGs content were measured. Non-decellularized matrices were used as a control. **Fig. S3.** In silico predictions for the binding site between miR-29-a/b/c and lncRNA *H19*. **Fig. S4.** Expression profile of *H19* during early osteogenic differentiation stages in MSCs from healthy donors (*N* = 4) and osteoporotic patients (*N* = 4). **Fig. S5.** miR-29c-3p expression after transfection of MSCs with miR-29c-3p mimics, miR-29c-3p inhibitor or respective controls (NC-mimics and NC-inhibitor), and cultured for 3 days in osteogenic-inducing conditions (*N* = 6). **Table SI.** Donors of Human Mesenchymal Stem/Stromal Cells. **Table SII.** Primers used for reverse transcription quantitative real-time PCR. **Table SIII.** Mature miRNAs sequences according to miRbase annotations (http://www.mirbase.org/). **Table SIV.** Antibodies used for immunocytochemistry stainings.

## Data Availability

The mass spectrometry proteomics data have been deposited to the ProteomeXchange Consortium via the PRIDE [62] partner repository with the dataset identifier PXD039001 and 10 .6019/PXD039001.

## Abbreviations

ALP: Alkaline phosphatase
AFM: Atomic force microscopy
CEBPA: CCAAT Enhancer Binding Protein Alpha
CEBPB: CCAAT Enhancer Binding Protein Beta
CTHRC1: Collagen triple helix repeat containing 1
COL1A1: Collagen Type I Alpha 1 Chain
COL1A2: Collagen Type I Alpha 2 Chain
COL 1: Collagen type I protein
COL3A1: Collagen Type III Alpha 1 Chain
DLX5: Distal-Less Homeobox 5
ECM: Extracellular matrix
FDR: False discovery rate
FBN1: Fibrillin-1
FE: Fold enrichment
FF: Fragility fractures
GO: Gene ontology
GAGs: Glycosaminoglycans
IBMX: 3-Isobutyl-1-methylxanthine
LDH: Lactate dehydrogenase
lncRNAs: Long non-coding RNAs
MSCs: Mesenchymal Stem/Stromal Cells
OA: Osteoarthritis
RFU: Relative fluorescence units
RUNX2: RUNX Family Transcription Factor 2
OPN: Secreted Phosphoprotein 1/Osteopontin
siH19: Small interfering RNA against H19
SOX9: SRY-Box Transcription Factor 9
TCPS: Tissue culture treated plastics
Tb.Sp: Trabecular separation
VTN: Vitronectin
