# Supplementary material for: Long non-coding RNA H19 regulates matrisome signature and impacts cell behavior on MSC-engineered extracellular matrices
Source: Stem Cell Res Ther. 2023 Mar 8;14:37. doi: 10.1186/s13287-023-03250-6 (PMC9993741; doi:10.1186/s13287-023-03250-6)
Supplement: Supplementary file 1 — Additional file 1: Fig. S1. Effect of siH19-2 on the expression levels of collagens, osteogenic markers and other H19-target candidates, assessed through RT-qPCR, 3 days after culture under osteogenic-inducing conditions (N = 6). Fig. S2. Decellularization of MSCs matrices. Primary MSCs from osteoporotic patients were differentiated for 10 days under osteogenic-inducing conditions before decellularization. After treatment with the decellularization buffer (urea 2 M), samples were stained with a) DAPI (blue) to visualize nuclei (scale: 100 μm) and b) hematoxylin (blue-purple color) to visualize nuclei and matrix integrity (scale: 100 μm). c) DNA and sGAGs content were measured. Non-decellularized matrices were used as a control. Fig. S3. In silico predictions for the binding site between miR-29-a/b/c and lncRNA H19. Fig. S4. Expression profile of H19 during early osteogenic differentiation stages in MSCs from healthy donors (N = 4) and osteoporotic patients (N = 4). Fig. S5. miR-29c-3p expression after transfection of MSCs with miR-29c-3p mimics, miR-29c-3p inhibitor or respective controls (NC-mimics and NC-inhibitor), and cultured for 3 days in osteogenic-inducing conditions (N = 6). Table SI. Donors of Human Mesenchymal Stem/Stromal Cells. Table SII. Primers used for reverse transcription quantitative real-time PCR. Table SIII. Mature miRNAs sequences according to miRbase annotations (http://www.mirbase.org/). Table SIV. Antibodies used for immunocytochemistry stainings. [file 13287_2023_3250_MOESM1_ESM.docx]

**Manuscript**

Long non-coding RNA H19 regulates matrisome signature and impacts cell behavior on MSC-engineered extracellular matrices

**Table of contents**

This document contains:

.Supplementary figures and respective legends

.Supplementary tables

.Supplementary methods

.Reference

**Supplementary figures and legends**


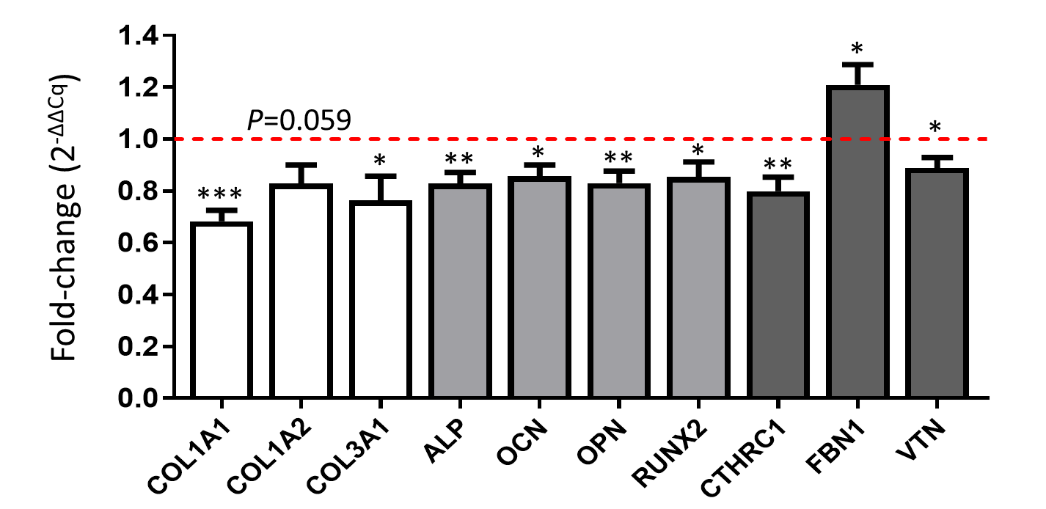


**Supplementary Figure 1.** Effect of siH19-2 on the expression levels of collagens, osteogenic markers and other H19-target candidates, assessed through RT-qPCR, 3 days after culture under osteogenic-inducing conditions (*N* = 6).

**
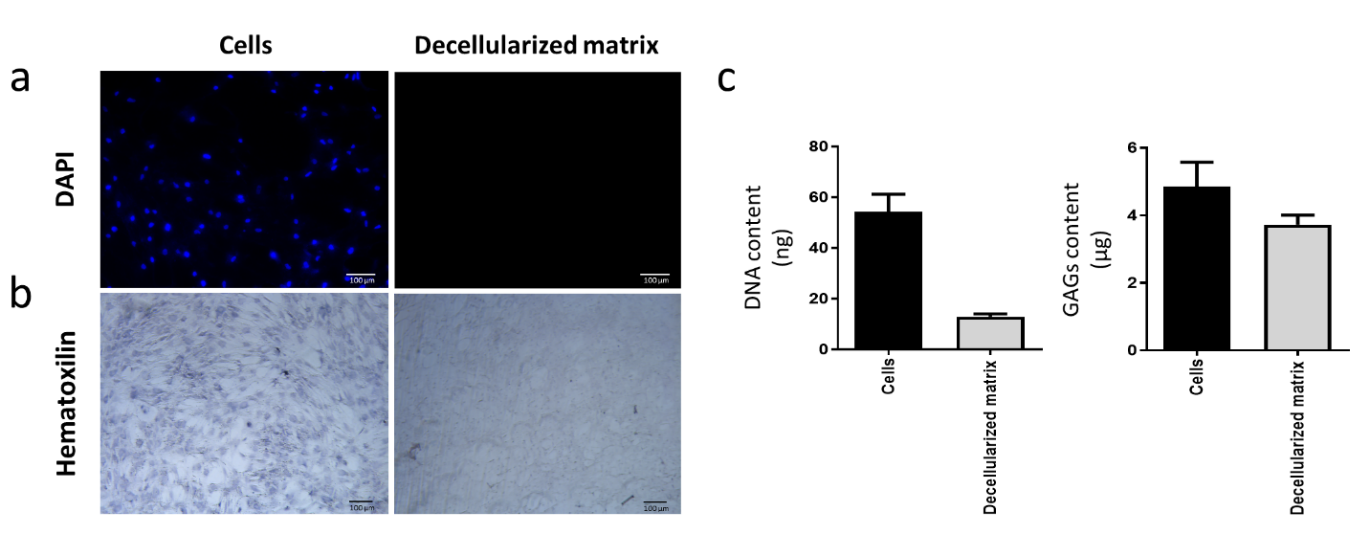
**

**Supplementary Figure 2.** Decellularization of MSCs matrices. Primary MSCs from osteoporotic patients were differentiated for 10 days under osteogenic-inducing conditions before decellularization. After treatment with decellularization buffer (urea 2M), samples were stained with a) DAPI (blue) to visualize nuclei (scale: 100 μm) and b) hematoxylin (blue-purple colour) to visualize nuclei and matrix integrity (scale: 100 μm). c) DNA and sGAGs content were measured. Non-decellularized matrices were used as a control.


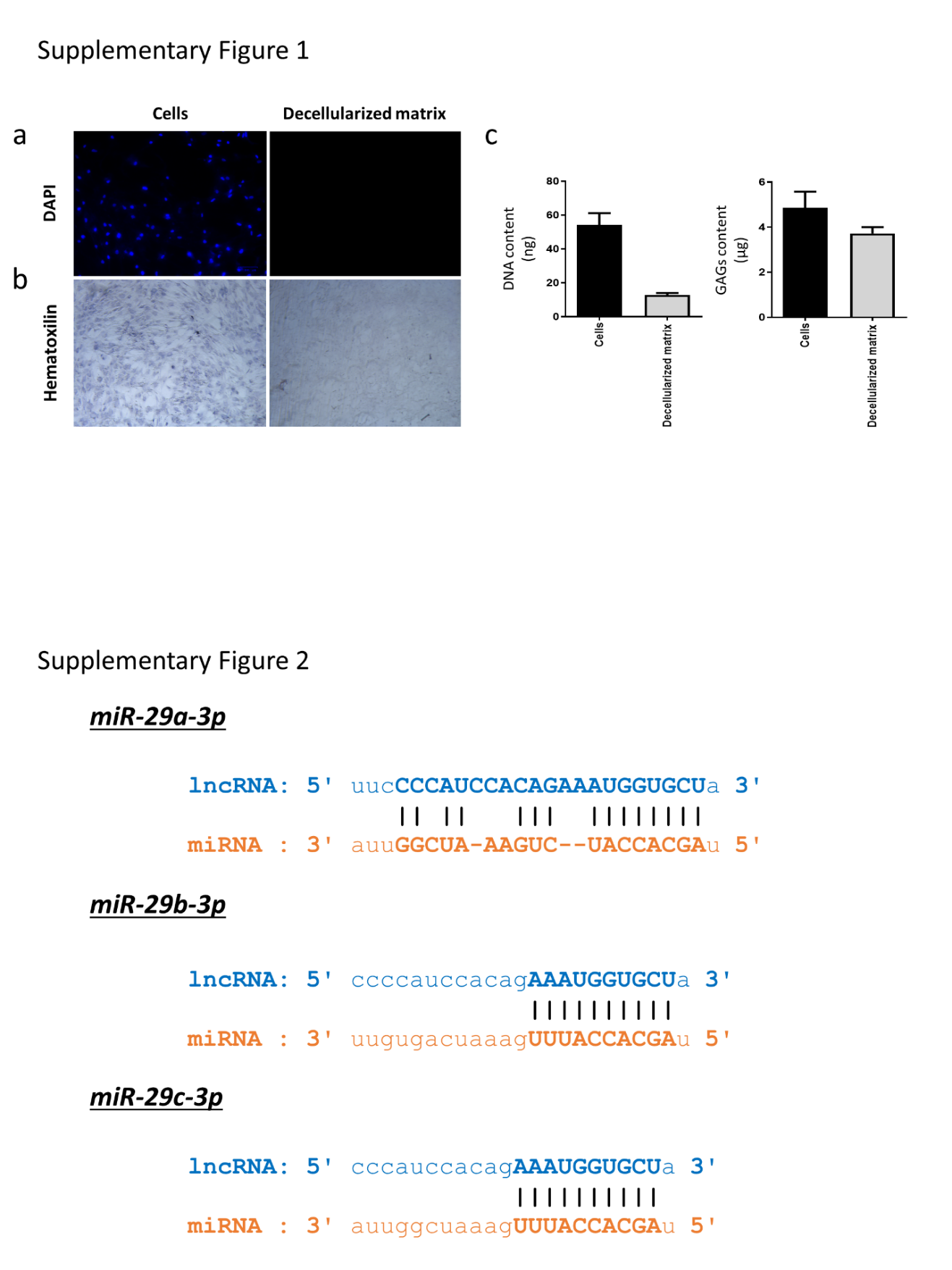


**Supplementary Figure 3.** *In silico* predictions for the binding site between miR-29-a/b/c and lncRNA *H19*.

**
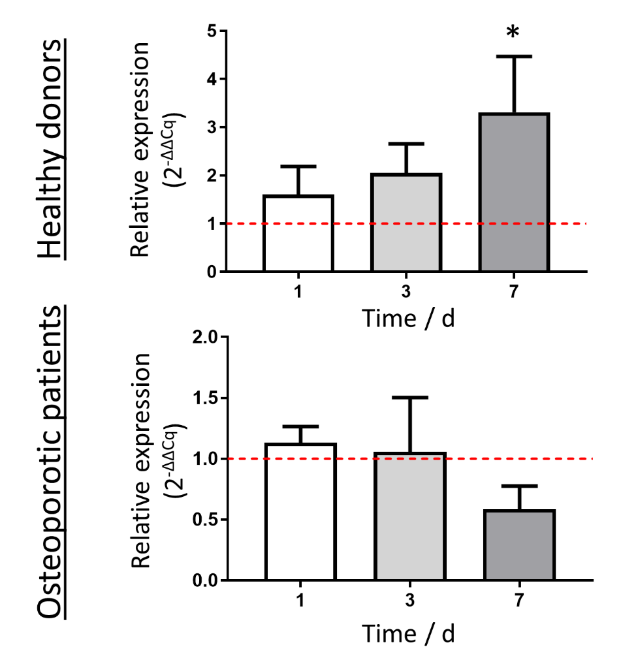
**

**Supplementary Figure 4.** Expression profile of *H19* during early osteogenic differentiation stages in MSCs from healthy donors (*N*=4) and osteoporotic patients (*N*=4).


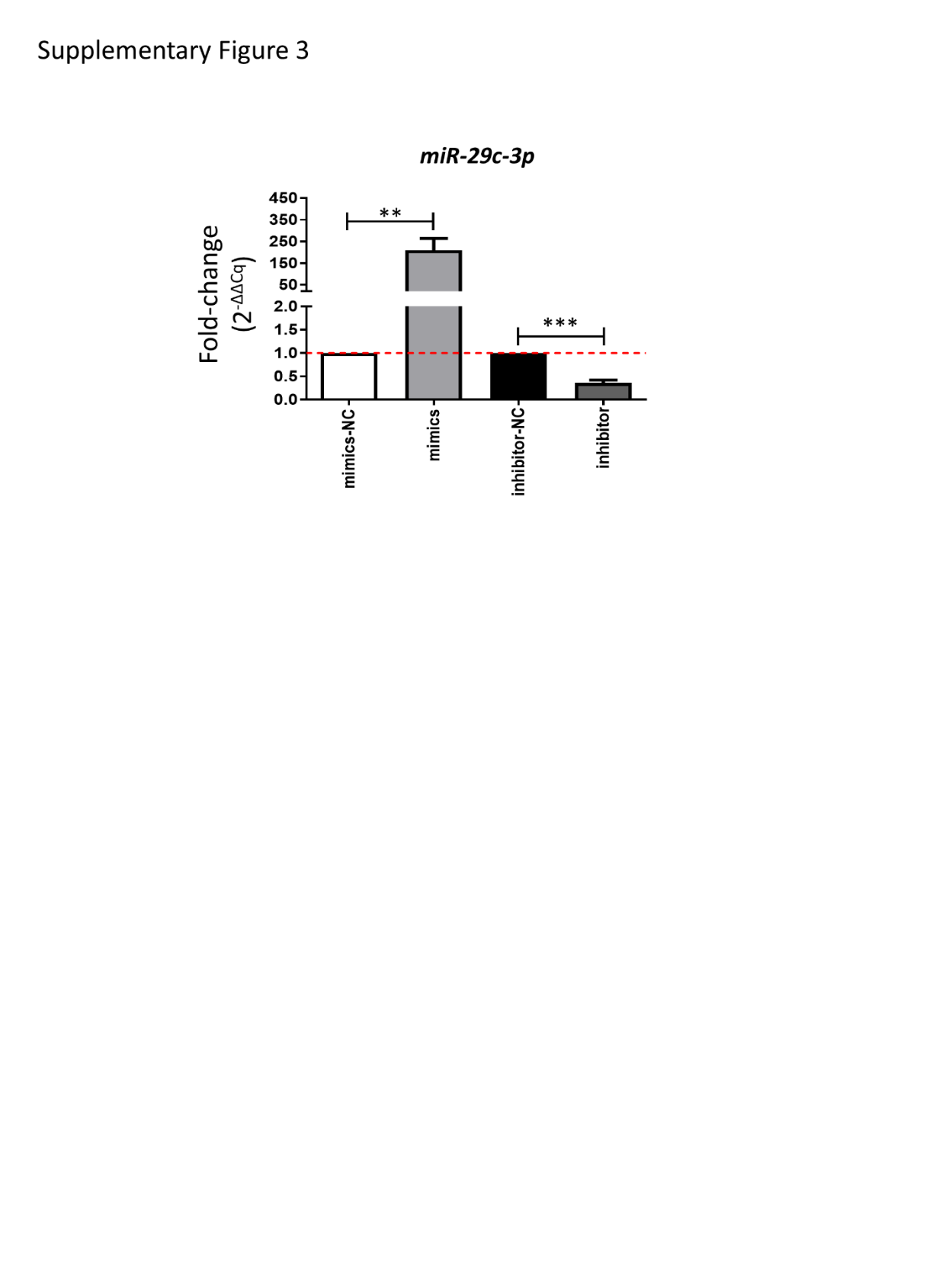


**Supplementary Figure 5.** miR-29c-3p expression after transfection of MSCs with miR-29c-3p mimics, miR-29c-3p inhibitor or respective controls (NC-mimics and
NC-inhibitor), and cultured for 3 days in osteogenic-inducing conditions (*N* = 6).

**Supplementary tables and legends**

**Supplemental Table I.** Donors of Human Mesenchymal Stem/Stromal Cells

|  | **Donor** | **Gender** | **Age (years)** | **Site of bone marrow collection** | **Surgery** |
| --- | --- | --- | --- | --- | --- |
| 1 | Osteoporotic | 1 | 80-89 | Femur | Cephalomedullary Nail |
| 2 | Osteoporotic | 1 | 70-79 | Femur | Hip arthroplasty |
| 3 | Osteoporotic | 1 | 70-79 | Femur | Hip arthroplasty |
| 4 | Osteoporotic | 2 | 70-79 | Femur | Hip arthroplasty |
| 5 | Osteoporotic | 2 | 70-79 | Femur | Hip arthroplasty |
| 6 | Osteoporotic | 2 | 90-99 | Femur | Hip arthroplasty |
| 7 | Healthy | 1 | 20-36 | Tibia | Anterior cruciate ligament injury |
| 8 | Healthy | 2 | 20-36 | Tibia | Anterior cruciate ligament injury |
| 9 | Healthy | 1 | 20-36 | Tibia | Anterior cruciate ligament injury |
| 10 | Healthy | 1 | 20-36 | Tibia | Anterior cruciate ligament injury |

**Supplementary Table II.** Primers used for reverse-transcription quantitative real-time PCR

| **Gene symbol** | **Definition** | **Origin** | **Primer sequence (5’ – 3’)** |
| --- | --- | --- | --- |
| ***ALPL*** | Alkaline phosphatase | Human | Fw: GACGGACCCGTCACTCTC |
|  |  |  | Rv: GTGCCCGTGGTCAATTCT |
| ***ACTB*** | Actin Beta | Human | Fw: TGGCACCCAGCACAATGAA |
|  |  |  | Rv: CTAAGTCATAGTCCGCCTAGAAGCA |
| ***CEBPA*** | CCAAT Enhancer Binding Protein Alpha | Human | Fw: ACTGGGACCCTCAGCCTTG |
|  |  |  | Rv: TGGACTGATCGTGCTTCGTG |
| ***CEBPB*** | CCAAT Enhancer Binding Protein Beta | Human | Fw: CGAAGTTGATGCAATCGGTTT |
|  |  |  | Rv: TTAAGCGATTACTCAGGGCCC |
| ***COL1A1*** | Collagen Type I Alpha 1 Chain | Human | Fw: GAGGGCCAAGACGAAGACA |
|  |  |  | Rv: CAGATCACGTCATCGCACA |
| ***COL1A2*** | Collagen Type I Alpha 2 Chain | Human | Fw: CCTGGTGCTAAAGGAGAAAGAGG |
|  |  |  | Rv: ATCACCACGACTTCCAGCAGGA |
| ***COL3A1*** | Collagen Type III Alpha 1 Chain | Human | Fw: GGAGCTGGCTACTTCTCGC |
|  |  |  | Rv: GGGAACATCCTCCTTCAAC |
| ***CTHRC1*** | Collagen Triple Helix Repeat Containing 1 | Human | Fw: CAATGGCATTCCGGGTACAC |
|  |  |  | Rv: CCAAGATCTATGCCATAATTCAA |
| ***DLX5*** | Distal-Less Homeobox 5 | Human | Fw: CCAACCAGCCAGAGAAAGAA |
|  |  |  | Rv: CCAACCAGCCAGAGAAAGAA |
| ***FBN1*** | Fibrillin 1 | Human | Fw: AGCGGAGCCGAGCAGTGG |
|  |  |  | Rv: GCTGCTCCCACTTCAGGC |
| ***GAPDH*** | Glyceraldehyde-3-Phosphate Dehydrogenase | Human | Fw: CCTCAAGATCATCAGCAAT |
|  |  |  | Rv: CCATCCACAGTCTTCTGGGT |
| ***H19*** | H19 Imprinted Maternally Expressed Transcript | Human | Fw: ACTGCACTACCTGACTCAGGAAT |
|  |  |  | Rv: AAGAGACAGAAGGATGAAAAAGA |
| ***MALAT1*** | Metastasis Associated Lung Adenocarcinoma Transcript 1 | Human | Fw: CCGAGCTGTGCGGTAGGCATT |
|  |  |  | Rv: CGGTTTCCTCAAGCTCCGCCT |
| ***OCN*** | Osteocalcin | Human | Fw: GGCGCTACCTGTATCAATGG |
|  |  |  | Rv: TCAGCCAACTCGTCACAGTC |
| ***OSX*** | Osterix | Human | Fw: GCCAGAAGCTGTGAAACCTC |
|  |  |  | Rv: GCTGCAAGCTCTCCATAACC |
| ***OPN*** | Osteopontin | Human | Fw: TCACCAGTCTGATGAGTCTCAC |
|  |  |  | Rv: CAGGTCTGCGAAACTTCTTAGAT |
| ***RUNX2*** | RUNX Family Transcription Factor 2 | Human | Fw: CCTGAACTCTGCACCAAGTC |
|  |  |  | Rv: GAGGTGGCAGTGTCATCATC |
| ***SOX9*** | SRY-Box Transcription Factor 9 | Human | Fw: TTCCTCCTGCCTTTGCTTGT |
|  |  |  | Rv: CGTGCTTGAAACATTCCCAGAAC |
| ***VTN*** | Vitronectin | Human | Fw: TGGCTGTCCTTGTTCTCCAGTG |
|  |  |  | Rv: GTGTGCGAAGATTGACTCGGTAG |
| ***XIST*** | X Inactive Specific Transcript | Human | Fw: AAGGATGTCAAAAGATCGGC |
|  |  |  | Rv: CAGCGTGGTATCTTCAATGG |

**Supplementary Table III.** Mature miRNAs sequences according to miRbase annotations (http://www.mirbase.org/)

| **miRNA** | **Acession** | **Mature sequence (5’ – 3’)** |
| --- | --- | --- |
| hsa-miR-29a-3p | MIMAT0000086 | UAGCACCAUCUGAAAUCGGUUA |
| hsa-miR-29b-3p | MIMAT0000100 | UAGCACCAUUUGAAAUCAGUGUU |
| hsa-miR-29c-3p | MIMAT0000681 | UAGCACCAUUUGAAAUCGGUUA |

**Supplementary Table IV.** Antibodies used for immunocytochemistry stainings

| **Antibody** | **Symbol** | **Reference** | **host** | **Dilution** | **Manufacturer** |
| --- | --- | --- | --- | --- | --- |
| Collagen type I | COLI | 600-401-103 | rabbit | 1:250 | Rockland |
| Osteopontin | OPN | sc-21742 | mouse | 1:250 | Santa Cruz Biotechnology |
| Fibrillin 1 | FBN1 | MA5-12770 | mouse | 1:100 | Thermo Fisher Scientific |
| Collagen Triple Helix Repeat Containing 1 | CTHRC1 | sc-293270 | mouse | 1:50 | Santa Cruz Biotechnology |
| Vitronectin | VTN | sc-74484 | mouse | 1:25 | Santa Cruz Biotechnology |
| Ki-67 | Ki-67 | MA5-14520 | rabbit | 1:100 | Thermo Fisher Scientific |
| IgG (H+L) Alexa Fluor ® 647 | N/A | A-21245 | rabbit | 1:1 000 | Thermo Fisher Scientific |
| IgG (H+L) Alexa Fluor ® 647 | N/A | A-31571 | mouse | 1:1 000 | Thermo Fisher Scientific |
| IgG (H+L) Alexa Fluor ® 488 | N/A | A-11001 | mouse | 1:1 000 | Thermo Fisher Scientific |

**Supplementary methods**

**1. Human primary bone marrow-derived MSCs culture**

Adherent isolated cells were cultured in monolayer with growth media (low-glucose Dulbecco's Modified Eagle's Medium (DMEM, Corning) supplemented with 10 % (v/v) fetal bovine serum (FBS, mesenchymal stem cell-qualified, Gibco) and 1 % (v/v) penicillin/streptomycin (P/S, Invitrogen) at 37 °C and 5 % (v/v) CO_2_. The media was changed twice a week and cells detached with trypsin-EDTA (Gibco) before reaching confluence. Isolated MSCs were characterized by flow cytometry and their potential to differentiate into osteogenic, adipogenic and chondrogenic lineage was tested. All MSCs used in the study followed the criteria described by the International Society for Cellular Therapy ^1^. Cells were maintained in culture up to eight passages maximum.

2. **Alkaline phosphatase (ALP) staining and quantification**

ALP staining was performed at day 7 of osteogenic differentiation. ALP solution was prepared by adding Naphthol AS-MX Phosphate alkaline solution (Sigma-Aldrich) to 0.25 g/L the Fast Violet filtered solution (Sigma-Aldrich). Cells were incubated with the ALP solution for 45 min, at RT, protected from the light. Following the removal of the staining solution, cells were washed twice with PBS 1x. Cells were then visualized under Leica DMi1 Inverted Microscope (Leica Microsystems). ALP intensity was quantified in 5 separate random fields of each well and expressed as the ratio between the area stained with ALP and the total analyzed area.

**3. Alizarin Red S staining and quantification**

Detection of calcium deposits and matrix mineralization was performed after 14 days in osteogenic-inducing conditions, through an Alizarin Red S staining. Cells were fixed with 40 g/L paraformaldehyde (PFA) for 30 min, at RT, washed twice with PBS 1x and incubated with freshly prepared 0.01 g/mL Alizarin Red S solution [Sigma-Aldrich, diluted in ethanol 2 % (v/v)] for 10 min, at RT, protected from the light. The staining solution was then removed, and the stained cells washed twice with PBS 1x. Cells were visualized under Leica DMi1 Inverted Microscope (Leica Microsystems). Alizarin intensity was quantified in 5 separate random fields of each well and expressed as the ratio between the area stained with Alizarin and the total analyzed area.

Alizarin Red S staining was solubilized by washing the cells several times with PBS 1x to remove non-specific staining. To elute the staining, a 0.1 g/mL cetylpyridinium chloride (CPC, Sigma-Aldrich) fresh solution was added to each well and incubated for 15 min, at RT and in constant rotation. The solubilized staining was collected, transferred to a 96 well-plate and measured at 562 nm using the spectrophotometer microplate reader Synergy MX (Biotek Synergy).

4. **Oil Red O staining**

The Oil Red O staining allowed for a qualitative analysis of adipocyte differentiation and to distinguish preadipocytes from adipocytes. The previously fixated cells were incubated for 5 min with 60 % (v/v) isopropanol (Sigma-Aldrich), rinsed and incubated with freshly prepared 3 g/L Oil Red O solution (Sigma-Aldrich), for 5 min, at RT. The staining solution was then removed, and the cells washed, until the water was running clear. Finally, formation of lipid droplets by the mature adipocytes (stained red) was acquired with the Leica DMi1 Inverted Microscope (Leica Microsystems).

Relative Oil Red O Accumulation was also measured by spectrophotometry. Briefly, the dye was eluted through the addition of isopropanol 100 % (v/v) and incubated for 15 min on a plate shaker, at RT. The samples were then transferred to 96 well-plates and the absorbance was read and measured at 540 nm, using the spectrophotometer microplate reader Synergy MX (Biotek Synergy).

**5. Proteomic analysis**

For proteomic analysis, 100 μg of protein was used. Protein identification and quantitation were performed using nanoLC-MS/MS, composed by an Ultimate 3000 liquid chromatography system coupled to a Q-Exactive Hybrid Quadrupole-Orbitrap mass spectrometer (Thermo Scientific). Samples were loaded onto a trapping cartridge for 3 min and further separated on an nano-C18 column at 300 nL/min. Peptide separation gradient was the following (A: 0.1 % (v/v) FA, B: 80 % (v/v) ACN 0.1 % (v/v)): 5 min (2.5 % (v/v) B to 10 % (v/v) B), 100 min (10 % (v/v) B to 35 % (v/v) B), 20 min (35 % (v/v) B to 55 % (v/v) B), 3 min (55 % (v/v) B to 99 % (v/v) B) and 12 min (hold 99 % (v/v) B). Data acquisition was controlled by Xcalibur and Tune software (Thermo Scientific).

The mass spectrometer was operated in data-dependent positive acquisition mode alternating between a full scan (m/z 380-1580) and subsequent HCD MS/MS of the 10 most intense peaks from full scan. Raw data were processed using Proteome Discoverer 2.5. software (Thermo Scientific). Protein identification was performed with Sequest HT search engine against the *Homo sapiens* entries from the UniProt database (https://www.uniprot.org/). Mass tolerance was 10 μg/g for precursor and 0.02 Da for fragment ions, respectively. Maximum allowed missing cleavage sites was set to 2. Cysteine carbamidomethylation was defined as constant modification. Methionine oxidation and protein N-terminus acetylation were defined as variable modifications. Protein and peptide confidence was set to high. The processing node Percolator was enabled with the following settings: maximum delta Cn 0.05; decoy database search target FDR 1 %, validation of based on q-value. Analysed samples were normalized against to the total peptide signal in each experiment and its quantitative evaluation was achieved by pairwise comparisons of the detected peptides. Data were corrected using Benjamin Hochberg method. Protein levels were compared by using the median ratio.

**Reference**

1. Dominici, M. *et al.* Minimal criteria for defining multipotent mesenchymal stromal cells. The International Society for Cellular Therapy position statement. *Cytotherapy* **8**, 315–317 (2006).
